# Supplementary figures and images for: A simple clinical score to reduce unnecessary testing for Puumala hantavirus
Source: PLoS One. 2024 May 31;19(5):e0304500. doi: 10.1371/journal.pone.0304500 (PMC11142550; doi:10.1371/journal.pone.0304500)

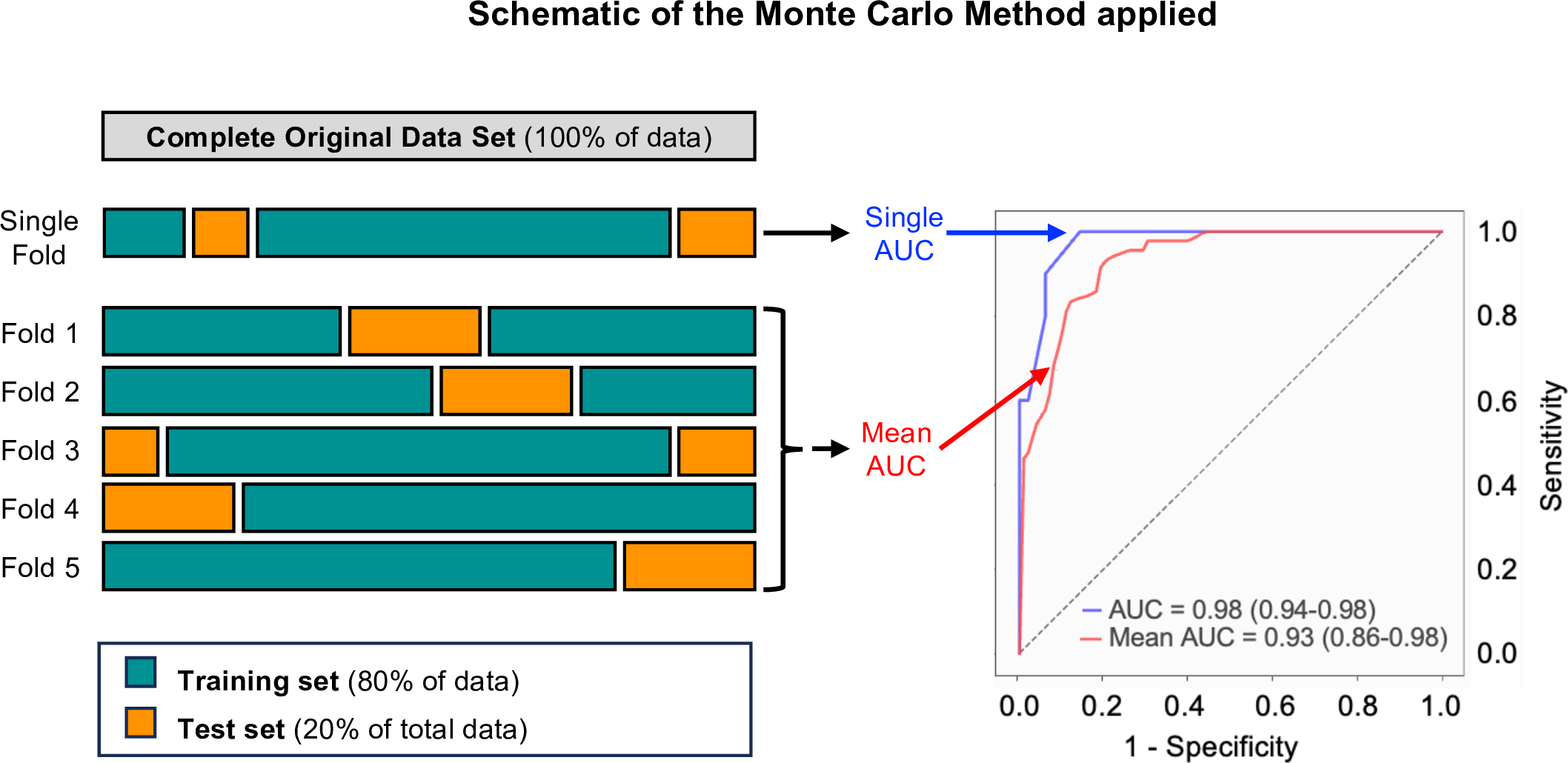

Supplement: S1 Fig — (TIF) [file pone.0304500.s001.tif]
