# Supplementary material for: A simple clinical score to reduce unnecessary testing for Puumala hantavirus
Source: PLoS One. 2024 May 31;19(5):e0304500. doi: 10.1371/journal.pone.0304500 (PMC11142550; doi:10.1371/journal.pone.0304500)
Supplement: S1 Table — (PDF) [file pone.0304500.s002.pdf]

**S1 Table. Comparison of potential scores with corresponding β-coefficients and the applied rounding**		**PUUV-pos. (N=30) vs. all PUUV-neg. (N=263)**			**PUUV-pos. (N=30) vs. PUUV-neg. viral (N=20)**		
		APS 1<br>4 criteria	5 criteria incl. PLT<150/nL	4 criteria with PLT<150/nL instead of AKI	APS 2<br>4 criteria	5 criteria incl. PLT<150/nL	4 criteria with PLT<150/nL instead of AKI
Point value	Fever	2.13	2.13	1.82	1.11	1.12	0.88
	Headache	2.26	2.18	2.08	1.15	1.16	1.21
	AKI	1.39	1.46	-	1.53	1.52	-
	LDH >300 U/L	1.21	1.11	1.16	1.36	1.38	1.57
	PLT<150 /nL	-	0.58	0.42	-	-0.07	-0.32
Rounded to	Fever	2	2	1	1	1	0
	Headache	2	2	2	1	1	1
	AKI	1	1	-	1	1	-
	LDH >300 U/L	1	1	1	1	1	1
	PLT<150 /nL	-	0	0	-	0	0

Rounding was performed to the lower positive number, e.g.: 2.13 to 2; 1.21 to 1; 0.58 to 0; 0.42 to 0; -0.32 to 0.

Abbreviations: AKI, acute kidney injury; APS, Aachen PUUV Score; PLT, platelets; LDH, lactate dehydrogenase.
