# Supplementary material for: A simple clinical score to reduce unnecessary testing for Puumala hantavirus
Source: PLoS One. 2024 May 31;19(5):e0304500. doi: 10.1371/journal.pone.0304500 (PMC11142550; doi:10.1371/journal.pone.0304500)
Supplement: S2 Table — (PDF) [file pone.0304500.s003.pdf]

S2 Table.

PUUV-positive-only validation cohort of N=41 (2001-2012)
Study population and admission parameters

**Parameter Values**	***Median (IQR)***	***N* available for analysis**
Age [years]	42 (31-53)	41
Blood pressure [mmHg]	132/80 (120/72-140/85)	26
WBC [/nL; 4.0–10.0]	8.4 (7.2-12.1)	41
Platelets [/nL; 150–400]	151 (111-268)	41
Hb females [g/dL; 12–16]	11 (10-11.9)	8
Hb males [g/dL; 14–18]	13.7 (12.9-15.3)	33
LDH [U/L; <250]	295 (269-354)	41
CRP [mg/L; <0.5]	31 (23-53)	41
Creatinine [mg/dL; 0.5–1.2]	3.3 (1.7-4.4)	41
Proteinuria [rank 0-6]	1 (0-4)	29
Hematuria [rank 0-6]	0 (0-2)	29
**Parameter Frequencies**	***N (%)***	***N* available for analysis**
Gender: male patients	33 (80%)	41
KDIGO AKI present	37 (90%)	41
WBC >10/nL	16 (39%)	41
Platelets <150/nL	20 (49%)	41
LDH >300 U/L	20 (49%)	41
CRP >100 mg/L	1 (2%)	41
Proteinuria	19 (66%)	29
Hematuria	14 (48%)	29
**Symptom Frequencies**	***N (%)***	***N* available for analysis**
Fever	40 (98%)	41
Headache	11 (27%)	41
Flu-like	15 (37%)	41
Loin Pain	19 (46%)	41
Gastrointestinal	18 (44%)	41
Edema	9 (22%)	41
Splenomegaly	6 (15%)	41
Conjunctivitis	1 (2%)	41
Petechiae	4 (10%)	41

Abbreviations: [ ], unit and reference range; AKI, acute kidney injury according to KIDIGO; CRP, C-reactive protein; Hb, hemoglobin; IQR, interquartile range; LDH, lactate dehydrogenase; N, absolute number of patients; PCT, procalcitonin; PUUV, acute Puumala hantavirus infection status; WBC, white blood count.
