# Supplementary material for: A simple clinical score to reduce unnecessary testing for Puumala hantavirus
Source: PLoS One. 2024 May 31;19(5):e0304500. doi: 10.1371/journal.pone.0304500 (PMC11142550; doi:10.1371/journal.pone.0304500)
Supplement: Attachment — Submitted filename: Point by point responses.pdf [file pone.0304500.s006.pdf]

Von: "PLOS ONE" <em@editorialmanager.com>
Betreff: PLOS ONE Decision: PONE-D-21-05086 - [EMID:4ab86222f83e97e6]
Datum: 5. März 2021 um 10:56:52 MEZ
An: "Gerald S Braun" <gbraun@ukaachen.de>
Antwort an: "PLOS ONE" <plosone@plos.org>

PONE-D-21-05086
A scoring system for the clinical pretest probability of Puumala hantavirus infection
PLOS ONE

Dear Prof. Braun,

Thank you for submitting your manuscript to PLOS ONE. After careful consideration, we have decided that your manuscript does not meet our criteria for publication and must therefore be rejected.

I am sorry that we cannot be more positive on this occasion, but hope that you appreciate the reasons for this decision.

Yours sincerely,

Prof. Dr. Joerg Latus
Academic Editor
PLOS ONE

Reviewer #1: Thank you for the opportunity to review the article by Braun et al. In their study, the researchers investigated hantavirus-positive patients to validate a previously published prediction score for acute renal failure. In another part, a new prediction score regarding the probability of positive hantavirus detection was developed based on clinical and serological parameters in positive and negative patients. The basic idea of the work is understandable and of value for clinical practice. However, the presented study shows some shortcomings, so I cannot recommend it for publication.
I will explain my points of criticism in the following:
Abstract: In the methods, both subprojects are described, but in the results, only the newly developed prediction tool is described.
In the methods section, the authors report that they used the "most frequent" method when data were missing. Here, additional information should be given on what percentage of data was missing, as this method is prone to error. Also, more detail should be provided on which data were missing and at what frequency.
In the first part of the study, the authors report a positive history of exposure in 70% of the cases in which the history was obtained. From my point of view, the question arises whether the positive history should not be part of the prediction score. The authors state that due to the retrospective character of the study many anamnesis data are missing, this is a clear limitation of the study and devalues it from my point of view.
In the discussion it is stated that for the authors a pretest probability of 25% is considered sufficient. However, it remains unclear whether this assessment is supported by other publications; the authors should comment on this.
In my opinion, another major limitation of the study is the very small number of patients with positive evidence in the second part of the study. In my opinion, the authors of the study should have put more effort into this and should also have evaluated the positive and negative tests for the years of the first part of the study in order to be able to make a stronger statement.
The biggest problem I see in the study presented is the over emphasis of symptoms in the prediction score. With the presence of headache and fever alone, the 4 points recommended for testing are reached. Since these two symptoms are certainly among the most common symptoms

in an emergency department, I see a very great danger of overdiagnosis here, the avoidance of which was actually a goal of the study. It would be interesting to know how often the described symptoms occur in combination in the emergency department of their own clinic and how often this would lead to hanta virus testing.

The authors reply to reviewer #1:

For an easier approach, first, we would like to organize the reviewer's criticisms into the following points:

1. Confusion for the reader created by the paper's seemingly arbitrary division into two sub-cohorts:
    a. a sample of N=79 hantavirus-positive individuals identified between 2001-2019 used to i) establish hallmark symptoms and to ii) validate a previously published "developing AKI" prediction-score
    b. a sample of N=165 individuals identified between 2016-2019 that had been subjected to serological testing for hantavirus and harnessed to derive a novel clinical prediction score for the probability of a positive hantavirus test result, intended to assist clinicians' testing decisions
2. Limited number of hantavirus-positive cases (N=21) in the second study. The reviewer stated that: *"the authors of the study should have put more effort into this and should also have evaluated the positive and negative tests for the years of the first part of the study in order to be able to make a stronger statement"*
3. Major issues:
    a. assertion in the former manuscript that a 25% positive predictive value be applied for clinical use of the score - thus suggesting an actual risk for over-testing
    b. questioning of the use of the common clinical symptoms of headache and fever in the score
    c. question why "history of exposure" was not included as a score-criterion
4. potential data loss (symptoms) from the retrospective review of patient histories
5. the reviewer requested more details on data losses filled in by "most frequent method"

The authors' point-by-point reply and changes to the manuscript:

1. The authors agree that the presentation of two sub-studies form diverging time-periods was highly confusing to the reader.

    The reason for this was not intentional - but due to issues of unavailable data for negatively tested cases when going further back in time.

    The elimination of study #1 (cohort of N=79 hanta positive cases from 2001-2019) and its corresponding analyses has now been undertaken.

    Only study #2 (cohort on cases subjected to hantavirus testing) represents the basis of the de-novo manuscript (see below for further details).

2. Following the suggestion made by the reviewer, we have now maximized the turnout of this study to the best of our possibilities from N=165 to N=293 individuals.

   This could be achieved since we were finally able to go back in time until 2013 to retrieve the comprehensive database of all performed hantavirus serologies. Unfortunately, we could not extend our analyses to prior to 2013, since identification of negatively tested subjects was unavailable given a change in the electronic documentation system 2012/13.

   Hence, we reperformed the score-generating analyses on a total of N=293 cases from 2013-mid 2021 and added an alternative approach by harnessing NE-negative subjects with proven viral infection as an alternative control group (novel Figure 4). Now, two scores were generated spanning the spectrum of real-world possibilities of how physicians approach those patients.

3. We agree with the reviewer that the choice of score elements (inclusion of fever, headache, low platelets, elevated lactate dehydrogenase - but omission of "history of exposure") and of our former threshold recommendations for clinical use (25% positive predictive value) merited reflection, modification and discussion:

   a. The study's main objective was and remains to provide a clinical estimation tool justifying the omission of serological hantavirus testing when the diagnosis is highly unlikely. In the de-novo version of the manuscript, in each score, a point sum of 0 or 1 translates into a pretest-probability for hantavirus positivity of 0%, meaning that this threshold allows for safe omission of testing (novel Figure 4).

      This is clinically relevant, since approximately half of the serological testing volume generated in our center belonged to this zero-probability group (novel Figure 4).

      Additionally, we have undertaken a partial (i.e. of the PUVV-positive arm) validation by using a historic collection of N=41 proven NE cases from 2001-2012 in which all 4 data points of the scores were reliably available of the 0-1 point sum criterium. Confirming our score application as a rule-out criterion no single case of NE exhibited 0 or 1 points but only higher ones.

   b. We took the criticism of the potentially high frequency in the Emergency Department of infections leading to headache / fever combinations very seriously. While we concede their non-specific character, according to our novel analyses, they still added meaningful value to our novel scores when it came to use for discounting a diagnosis of NE. This is also supported by a case-control study described in our novel discussion section.

      In our view, this is also supported by reasoning. NE is almost invariably associated with a substantially severe systemic illness-syndrome. This practically invariably involves a temperature of 39°C or higher. Indeed, in our study the threshold for "fever" is set to 39°C. As shown in Figure 3A, the frequency of this kind of fever was 90 vs. 30% and the frequency of headache was 70 vs.10% in NE versus hanta-negatively tested subjects, respectively.

      While AKI is now included into our novel scores since a score should contain its most defining hallmark laboratory-changes, it is implicit that the score be employed in conjunction with sound clinical reasoning.

      This means in the case of differential diagnosis in the emergency department for viral illnesses that: presence of typical organ tropisms such as to the respiratory, or gastrointestinal tract excludes NE / acute PUUV infection at the same time. The scores presented herein should be applied in this light. They are not an algorithm

for non-reflected semi-automatic differential diagnostic calculation of the PUUV status.

Estimating the co-incidence of fever and headache in our ED in a retrospective manner in a digital way was – unfortunately – impossible and not amenable to a manual nonspecific chart review. However, given the inferences made above, this point, in view of the authors, has lost a degree of its relevance.

c. The information from the former manuscript that 70% of NE cases may feature a positive history of exposure has been removed.

For one, it was based on a small selective sub analysis of study #1 - which is also no longer past of the manuscript.

Second, in our general clinical practice with NE, we do not find obtaining a "history of exposure" very fruitful in guiding the consideration of NE since it is usually both vague and prone to overinterpretation – thus precluding it in our view from inclusion into a score.

4. The authors agree that loss of data on patient histories and symptoms is a potential issue weakening the study. However, we hope that the reviewer agrees with us that i) the selection of symptoms relevant to our scores and ii) the high scrutiny applied in our study to retrieve the maximum of data by one-by-one chart review and by excluding all patients with incomplete clinical information / charts serves to handle such drawbacks appropriately. As laid out in the discussion section, comparison with the literature suggests a reliable data collection.
5. We estimate the potential bias ensuing from application of the most-frequent method in our hands as comparatively low: out of the four score-generating parameters fever, headache, AKI and LDH, only the latter criterion featured incomplete data sets at a low rate: 2/30 (6,7%) for PUVV-positive and 3/263 (1,1%) for PUVV-negative cases, respectively.

The authors would like to thank the reviewer for the remark that: "The basic idea of the work is understandable and of value for clinical practice."

In this light, the criticism presented by the reviewer was a main source of motivation to "put more effort in the evaluation of additional years of the hanta positive / negative serology cohort".

In addition, the graphical presentation was modified with to hopefully increase the data's intelligibility for the reader throughout the manuscript.

Reviewer #2: Thanks a lot for chosen me as a Reviewer for this manuscript:

In the current paper, Brockmann et al. conducted a two-step retrospective observational study on patients with nephropathia epidemica (NE) in the border Triangle Germany - Belgium and the Netherlands.
In a first step baseline characteristics of patients with positive Puumala hantavirus (PUUV) serology (period 2001 to 2019) were analyzed. Data were used validate an established clinical score published in 2015 by Latus et al. to predict severity of acute kidney injury (AKI) in NE.
In a second sub study all patients tested in 2016 to 2019 for PUUV serology were analyzed. Clinical and laboratory data at hospital admission were used to create a score to predict pretest probability for positive PUUV-Serology.

The goal of the current paper was to create a score for the prediction of the clinical probability of acute hantavirus infection. Here, all patients testing positively and negatively (2016-2019) for PUUV were directly compared and logistic regression analysis was performed. The first part of the paper aimed to compare the cohort of patients with NE with a previously published cohort of patients from south-western Germany. Additionally the already mentioned score for prediction AKI in patients with NE published by Latus et al. was validated in this 79 patients.

In principle the aim of the study (create a score for prediction of clinical probability of acute hantavirus infection) is interestingly not only for specialists in NE.

Though, I have a few comments.

1. Why do you used two different time periods of patients with positive PUUV-serology (time period 1 from 2001 to 2019 was used for validation a AKI-risk-score and time period 2 from 2016 to 2019 was used to create a new score for the prediction of the clinical probability of acute hantavirus infection) for analyzing your cohort of patients ?

2. In time period-1 65 patients out of 79 (84%) had have already an AKI at hospital admission (median creatinine at admission 2.3 (IQ 1.4-4.4). In my understanding an AKI-risk score cannot validate in a cohort of patients with already existing AKI. Please specify your methods to create the score you presented in figure S2.

3. Headline page 12:
"Study two – Comparison of 2016-2016 positive and negative patients", You mean comparison of 2016-2019 (time period 2) ?

4. Study two (Comparison of patients with thrombocytopenia admitted to hospital in 2016-2019): Main message of the second part is: "identification of patients with NE is important because AKI in NE is often severe but in nearly all cases self-limiting". A rapid identification of patients with NE in a cohort of patients classically in patients with AKI and thrombocytopenia is mandatory to identify patients with important differential diagnoses (e.g. thrombotic microangiopathy, sepsis or leptospirosis) with need of admission to intensive care unit. In patients with NE (21 participants) kidney function at hospital admission was impaired in most patients (median creatinine 2,0mg/dl, IQR 1,2 - 3,6) and platelets were decreased to a median of 79/nl (IQR 67-186).

Interestingly in the cohort of patients tested negative for PUUV platelets was normal in most patients (median platelets count 210 (124-284) but were suspected for AKI.

Depending on your clinical findings in 21 patients with NE you designed a score to predict probability of positive PUUV-serology in an emergency department (ED) cohort. Due to his nature as a systemic viral infection patients in NE have often clinical signs of a systemic virus infection for example fever and headache.

Your score highlighted the two clinical findings fever and headache each with 2 points. The two lab value parameters were evaluated each with one point. The pretest probability in your study was 25% for positive PUUV-serology in patients with fever and headache. Even though you performed polished statistical methods the groups you investigated was not optimal. As shown in figure 3 and S3 your control group was a colorful mixture of patients with sepsis, malignancy or systemic vasculitis. In my opinion you should compare the NE patients with patients that have a systemic virus infection as well if you want distinguish patients with NE in absence of AKI and thrombocytopenia. Around 16 patients out of 144 (13%) had have another viral infection. Another variant would be the analysis of all patients with AKI and low platelets. Otherwise you tested all patients with fever and headache for PUUV. Though your cohort of 21 patients with NE is very small.

I think the aim of your study should be to characterize all available patients with NE in your center (e.g. study-periode 2001 to 2019). With this data you should analyze all patients with AKI and thrombocytopenia to design a score to predict positive PUUV-Serology in for example a control group of patients with AKI and low platelets as well (perhaps matched for severity of AKI and thrombocytopenia at hospital admission). In a second step you could perform a prospective validation of the score int ED.

In summary I cannot recommend this paper for publication in PlosOne.

The authors reply to reviewer #2:

1. The issues raised here are identical with the ones raised by reviewer #1 under 1. and 2. Please find the see answers above.
2. Obsolete since the data is no longer part of the manuscript.
3. Yes - addressed.
4. **In this section, the reviewer commented extensively on study #2. Prior to providing our answers, we would like to reorder the remarks:**
    - paragraph 4: NE patients should also be compared to a subgroup with systemic viral infection alone
    - paragraph 5: suggestion of an alternative study design:
        - group A: all patients featuring the combination of NE, AKI and thrombocytopenia
        - group B: matched controls with AKI and thrombocytopenia
        - followed by a prospective validation study in the emergency department
    - paragraph 3: fever and headache are simply signs of the systemic viral infection present with hantavirus, yet they were used to design a score for PUUV / NE
    - paragraph 2: while AKI was common, admission platelet count was normal in the majority of hantavirus negatives

The authors' point-by-point reply and changes to the manuscript:

In light of the thoughtful and constructive comments provided to us, we have now reframed our study's goal, and its presentation and practical conclusions throughout the manuscript.

- paragraph 4: we thank the reviewer and have now included a second score devised by using a control group of confirmed viral infections
- paragraph 5: The study's main objective was and is to provide an estimation tool to justify the omission of serological testing for hantavirus when the diagnosis is highly unlikely. To this end, we have now performed novel analyses deriving two instead of one score(s). In each score, a point sum of 0 or 1 translated into a pretest-probability for hantavirus positivity of 0%. This was also now validated by an alternative independent NE-cohort of N=41 which exhibited no case of point sum 0 or one.

  It was and is not the study's objective to sensitively or to specifically diagnose NE in a non-selected a general population of Emergency Department patients. For such an end, indeed the study design suggested by the reviewer would be required, e. g. to identify AKI + low platelet + in NE combinations and to compare their characteristics to those of a matched control group of patients that does not need to sport a confirmed negative PUUV serology.

  This, however, was not our intention since NE has a low frequency in the ED rendering such an endeavor both unnecessary and unrealistic.

  In addition, we have now included an analysis on the testing results and behavior of the involved medical specialties showing that i) the diagnostic hit rate for a PUUV-positive subject was 3-fold higher in the department of nephrology compared to other medical specialties while at the same time ii) nephrologists were as prone to over testing of zero-probability patients as other medical specialists (novel Table 3).
- paragraph 3: the issue of the inclusion of the non-specific clinical symptoms of fever and headache into the score has also been commented on by reviewer #1.

  In our view, it is implicit that the improved scores presented herein be employed in conjunction with sound clinical reasoning. Outline and performance of our scores lie in the nature that they were based on the entirety of serologically tested individuals.

  This pre-selection implicitly defines the cohort as one pre-selected for PUUV testing that reflects clinical reasoning and practice of a heterogeneous group of physicians at our center - but (obviously) not necessarily the best and logical application of clinical reasoning and available knowledge on the differential diagnostic criteria of NE. Yet, we believe that this real-world-based approach also has its merits since it is rooted in what physicians do instead of what we would like them to do.

  In addition, as outlined in the discussion, one study came to very similar conclusions on the high discriminatory power of fever, headache and LDH elevation.
- paragraph 2: The frequency at the timepoint of admission of AKI (present in 75% of both groups; novel Figure 3B) was a much higher than for low platelets (60% in PUUV positives and 30% in PUUV negatives; novel Figure B).

  In our view, the explanation for this finding easy and not surprising

  - though thrombocytopenia is an invariable feature of NE, given its transient nature, an important proportion of NE patients (40% in our study) seek medical attention at a point where platelet counts have already recovered in NE

- simultaneously, AKI has a known longer time lag in NE
- clinicians – at least in our center – did not solely base their decision to suspect NE upon detection of the combination of AKI and low platelets, but also on either one of them
- taken together, we used this information to substitute the low platelets in the previous score by presence of AKI in the revised versions
